# Supplementary material for: Digital Cytology in Veterinary Education: A Comprehensive Survey of Its Application and Perception among Undergraduate and Postgraduate Students
Source: Animals (Basel). 2024 May 24;14(11):1561. doi: 10.3390/ani14111561 (PMC11170988; doi:10.3390/ani14111561)
Supplement: Supplementary file 1 [file animals-14-01561-s001.zip › animals-3002136-supplementary.pdf]

Table S1: Table 1 shows the 11 multiple-choice questions in the survey (HiDS = Histological digital slides; CDS = Cytological digital slides; HeDS = Hematological digital slides; WSI = Whole slide imaging).

**1. Position/Title** (choose one answer)
a. Veterinary Medicine Student
b. General practitioner
c. Post graduate student (e.g. PhD student, Postdoctoral fellow)
d. Clinical pathology resident
e. Anatomic pathology resident
f. Resident in other discipline
g. Other (please specify)
**2. Group age** (choose one answer)
a. 19-25 years
b. 26-35 years
c. 36-50 years
d. >50 years
**3. How many courses with glass slides have you attended?** (choose one answer)
a. 0
b. 1-3
c. 4-5
d. >5
**4. How many courses with digital slides have you attended?** (choose one answer)
a. 0
b. 1-3
c. 4-5
d. >5
**5. What types of digital slide have you used in the courses?**
a) HiDS
b) CDS
c) HeDS
d) HiDS and CDS
e) HiDS and HeDS
f) CDS and HeDS
g) HiDS and CDS and HeDS
**6. How were digital cytology cases presented during courses?** (choose one answer)
a. Pictures of selected areas of a cytology glass slides (static DP)
b. Robotic optical microscope with a camera remotely controlled by the teacher (dynamic DP)
c. WSI
d. Pictures of selected areas of a cytology glass slides and robotic optical microscope with a camera remotely controlled by the teacher (static and dynamic DP)
e. Pictures of selected areas of a cytology glass slides and robotic optical microscope with a camera remotely controlled by the teacher and WSI (static and dynamic DP and WSI)
f. Pictures of selected areas of a cytology glass slides and WSI (static and WSI)
g. Robotic optical microscope with a camera remotely controlled by the teacher and WSI (dynamic and WSI)
**7. Which is your favourite way to learn cytology?** (choose one answer)
a. Picture based cases
b. Glass cytology slides

c.	Digital cytology slides
d.	Robotic optical microscope with a camera remotely controlled by the teacher
e.	Only one of these methods used, therefore unable to compare and express a preference
f.	Other (please specify)
**8. What do you like the most about digital cytology case?** (choose one or more answers)	
a.	Freedom of navigating throughout the digital slide
b.	No time limit
c.	Limited equipment needed (no microscope)
d.	Possible to access digital cases when I want/need
e.	You're sure that you're looking at the same structures as your teacher
f.	Possible to discuss together of what we see
**9. What do you dislike about digital cytology cases?** (choose one or more answers)	
a.	Lack of guidance when navigating throughout the digital slide
b.	Technical problems in using the dedicated software to visualise digital slides
c.	Longer learning curve to adjust to digital slide assessment
d.	Inadequate resolution and/or different colours compared with glass slides or microscopic pictures
e.	Other (please specify)
**10. Did training with digital cytology help you to improve your cytology skills?** (choose one answer)	
a.	Yes
b.	No
**11. Have you learned more through digital cytology compared to the traditional courses with glass slides?** (choose one answer)	
a.	I have never attended any "traditional cytology course" before
b.	I prefer "traditional cytology courses"
c.	I prefer "traditional cytology courses" but integration with digital cytology is fine too
d.	Digital cytology is better than traditional cytology for learning purposes
**12. Other comments?** (please specify)	
